# Longitudinal association between change in the neighbourhood built environment and the wellbeing of local residents in deprived areas: an observational study

**DOI:** 10.1186/s12889-018-5459-9

**Published:** 2018-04-24

**Authors:** Louise Foley, Emma Coombes, Dan Hayman, David Humphreys, Andrew Jones, Richard Mitchell, David Ogilvie

**Affiliations:** 1 MRC Epidemiology Unit & UKCRC Centre for Diet and Activity Research (CEDAR), School of Clinical Medicine, University of Cambridge, Box 285 Institute of Metabolic Science, Cambridge Biomedical Campus, Cambridge, CB2 0QQ UK; 2 Norwich Medical School and CEDAR, University of East Anglia, Norwich, NR4 7TJ UK; 3 Unaffiliated, Cambridge, UK; 4 Department of Social Policy and Intervention, University of Oxford, Barnett House, 32 Wellington Square, Oxford, OX1 2ER UK; 5 MRC/CSO Social and Public Health Sciences Unit and Centre for Research on Environment, Society and Health, University of Glasgow, 200 Renfield Street, Glasgow, G2 3QB UK

**Keywords:** Built environment, Neighbourhood, Wellbeing, Natural experimental study

## Abstract

**Background:**

Features of the urban neighbourhood influence the physical, social and mental wellbeing of residents and communities. We explored the longitudinal association between change to the neighbourhood built environment and the wellbeing of local residents in deprived areas of Glasgow, Scotland.

**Methods:**

A cohort of residents (*n* = 365; mean age 50 years; 44% male; 4.1% of the 9000 mailed surveys at baseline) responded to a postal survey in 2005 and 2013. Wellbeing was assessed with the mental (MCS-8) and physical (PCS-8) components of the SF-8 scale. We developed software to aid identification of visible changes in satellite imagery occurring over time. We then used a Geographical Information System to calculate the percentage change in the built environment occurring within an 800 m buffer of each participant’s home.

**Results:**

The median change in the neighbourhood built environment was 3% (interquartile range 6%). In the whole sample, physical wellbeing declined by 1.5 units on average, and mental wellbeing increased by 0.9 units, over time. In multivariable linear regression analyses, participants living in neighbourhoods with a greater amount of change in the built environment (unit change = 1%) experienced significantly reduced physical (PCS-8: -0.13, 95% CI -0.26 to 0.00) and mental (MCS-8: -0.16, 95% CI -0.31 to − 0.02) wellbeing over time compared to those living in neighbourhoods with less change. For mental wellbeing, a significant interaction by baseline perception of financial strain indicated a larger reduction in those experiencing greater financial strain (MCS-8: -0.22, 95% CI -0.39 to − 0.06). However, this relationship was reversed in those experiencing lower financial strain, whereby living in neighbourhoods with a greater amount of change was associated with significantly improved mental wellbeing over time (MCS-8: 0.38, 95% CI 0.04 to 0.72).

**Conclusions:**

Overall, we found some evidence that living in neighbourhoods experiencing higher levels of physical change worsened wellbeing in local residents. However, we found a stronger negative relationship in those with lower financial security and a positive relationship in those with higher financial security. This is one of few studies exploring the longitudinal relationship between the environment and health.

**Electronic supplementary material:**

The online version of this article (10.1186/s12889-018-5459-9) contains supplementary material, which is available to authorized users.

## Background

The majority of the world’s population now live in urban areas, with the proportion projected to increase to two thirds of the population by 2050 [1]. An accumulating body of work indicates that the urban neighbourhood can influence the physical, social and mental wellbeing of residents and communities [2]. Together, this suggests that urban design has a key role to play in supporting population health in the future [3].

However, substantial inequalities in health exist between the least and most deprived neighbourhoods in cities [1]. It has been proposed that in deprived neighbourhoods, multiple features of the physical environment [4] (for example, pollution, lack of access to transport, shops or health care, as well as incivilities such as litter and poorly maintained pavements) could compound to exacerbate health inequalities – a concept termed ‘deprivation amplification’ [5]. The relationship between deprivation and ill health is not straightforward and appears to interact with a complex arrangement of individual and ecological factors [6]. For example, efforts to tease out the relative contributions of area- and individual-level socioeconomic circumstances on heath suggest that low socio-economic status individuals living in deprived areas tend to have the worst health outcomes [7, 8], whereas higher socio-economic status can buffer individuals from the negative effects of living in deprived areas [8].

Urban renewal or regeneration can take many forms, but is often associated with changes to the physical built environment. This includes activities such as the creation or removal of parks, the construction of community centres, and upgrades or repairs to properties or streets, as well as the demolition of existing housing stock or other community amenities. Urban renewal is proposed as one way to target the wider physical, social and economic determinants of ill health in deprived communities [9]. Some evidence suggests that urban renewal can promote modest improvements in health and wellbeing [9–12], and reduce, or at least not exacerbate, health inequalities [9, 12–14]. However, the evidence base is far from conclusive.

Previously we demonstrated that exposure to one specific type of major change to the urban environment (motorway construction) was associated with detrimental changes to wellbeing in local residents [15]. Prior to its construction, the motorway was described by supporters as both a component of, and a catalyst for, urban renewal [16]. Here, we consider general change occurring in the built environment, and explore the feasibility of using a novel and simple method to quantify the degree of change occurring over time (though not characterising the changes). Our specific aims were to: (a) examine the longitudinal association between exposure to general change in the neighbourhood built environment and change in wellbeing; (b) explore whether this relationship differed in population sub-groups; and (c) explore whether this relationship differed in those additionally exposed to a specific change in the built environment, a new urban motorway, not captured by the primary exposure measure.

## Methods

### Context

Glasgow (593,200 inhabitants in Glasgow City) [17] is the largest city in Scotland and the fourth largest city in the United Kingdom (UK). Glasgow has the lowest life-expectancy in the UK [18] and is characterised by extremes of affluence and deprivation [19].

### Design

We examined the relationship between change in the neighbourhood built environment and wellbeing in a longitudinal cohort recruited at baseline (2005) and follow-up (2013). The analyses reported here are part of a larger mixed-method natural experimental study examining the effects of a new urban motorway on travel, physical activity, road traffic accidents and wellbeing in local communities. The study received ethical approval from the University of Glasgow (baseline reference FM01304; follow-up reference 400120077).

Further information on the methods and findings of the baseline [16, 20] and follow-up [15, 21–23] study can be found elsewhere.

### Study areas

**Fig. 1:**
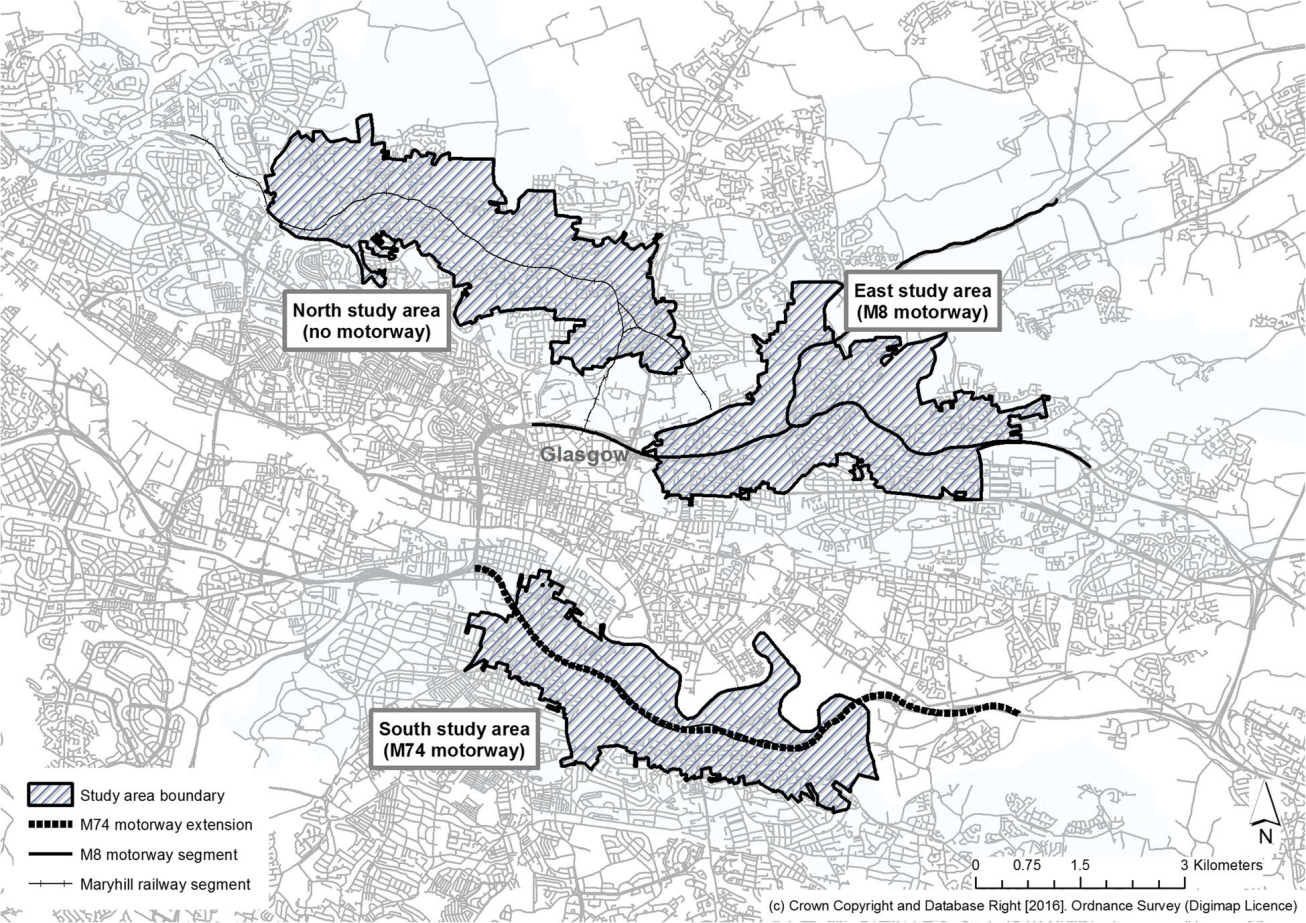
Boundaries of original study areas in Glasgow, Scotland. Crown Copyright and Database Right [2016]. Ordnance Survey (Digimap Licence)

The overall study aimed to examine how living near an urban motorway affected activity and health outcomes. At baseline we delineated three study areas in Glasgow using a Geographic Information System (GIS). Using spatially referenced census data combined with field visits, we created study areas which were broadly comparable on key socioeconomic (area deprivation, housing tenure, car access) and topographic (land use mix, arterial road network, distance from city centre) factors [16]. The three areas differed by proximity to a motorway; the South study area contained a new motorway (the M74), the East study area contained an existing motorway (the M8) and the North study area contained a railway segment but no motorway infrastructure (Fig. 1).

Because of the comparability of the three areas, and because we found no baseline sociodemographic differences by study area [20], for the purpose of this analysis we combined the three study areas.

### Sampling and recruitment of participants

At baseline in 2005, we recruited an initial sample of adults aged 16 years or over using a postal survey delivered to a random sample of private residential addresses in each of the three study areas. The sample was drawn from the Royal Mail Postcode Address File and 9000 surveys in total were mailed (3000 in each study area). Participants were informed that return of the survey constituted consent to participate in the study. Respondents were also given the option to consent to future contact, and yearly contact (in the form of a greeting card) was maintained with those who agreed. At follow-up in 2013, those who could still be contacted were mailed another survey. While those who were known to have moved out of the study areas (*n* = 14) or Glasgow (*n* = 8) were followed up if they remained in the UK, they were removed for the purpose of this analysis as we could not generate an exposure measure for these individuals.

To maximise our response and buffer against likely attrition over the 8 year follow-up period, we followed recommendations for the use of postal surveys [24] including sending notification postcards prior to the main survey, following up non-response, and offering a small monetary incentive (£50 prize draw at baseline and £5 voucher at follow-up). At both time points, the survey was mailed in October and staggered across multiple days, and responses received more than 3 months after the first mailing were disregarded.

### Measurement

The survey included items on demographic and socioeconomic characteristics, travel behaviour, physical activity and wellbeing. Further detail on the assessment of wellbeing, and the covariates used in the models, is given below.

#### Wellbeing

At both baseline and follow-up, wellbeing was assessed using the SF-8 [25], an eight item scale evaluating health status in the previous month. Items were scored on 5- or 6-point Likert scales, with physical and mental component summary scores (PCS-8 and MCS-8, respectively) then derived using standard procedures [25]. Higher scores on these scales reflect greater wellbeing. The SF-8 has been extensively validated and shown to be sensitive to change in the health status of the individual [25]. The scale is highly correlated with the original 36 item version (SF-36) [26] which has been shown to have associations with job loss, primary care utilisation and five-year survival [26].

#### Covariates

At both baseline and follow-up, participants reported their age in years, sex, housing tenure, number of cars owned, working status, perceived financial strain, whether they had a chronic condition and years lived in the local area. For the purpose of analysis, housing tenure was dichotomised as owner-occupier versus other, car ownership as not owning a car versus owning at least one car and working status as working or studying versus other. Perceived financial strain was assessed with the following item: “Thinking about the cost of living as it affects you and your household, which of these best describes your situation at present?” The response options were: “Find it a strain to get by from week to week”; “Have to be careful about money”; “Able to manage without much difficulty”; and “Quite comfortably off”. For the purpose of analysis, this was dichotomised as more strain (combining the options “Have to be careful with money” and “Find it a strain to get by”) or less strain (combining the options “Can manage without difficulty” and “Quite comfortably off”).

#### Exposure to change in the neighbourhood built environment

We wished to quantify the degree of physical change occurring in participants’ local area over the course of the study, in order to investigate how exposure to such change was related to wellbeing. However, the UK does not have off-the-shelf data with which to describe built environment change over time. Current and historical satellite imagery is freely available and covers most of the globe, including the study areas. Therefore, we explored the possibility of using Google Earth as a source of information on change to the built environment over time. This complements work by others using Google products such as Street View to explore aspects of the built environment [27, 28].

**Fig. 2:**
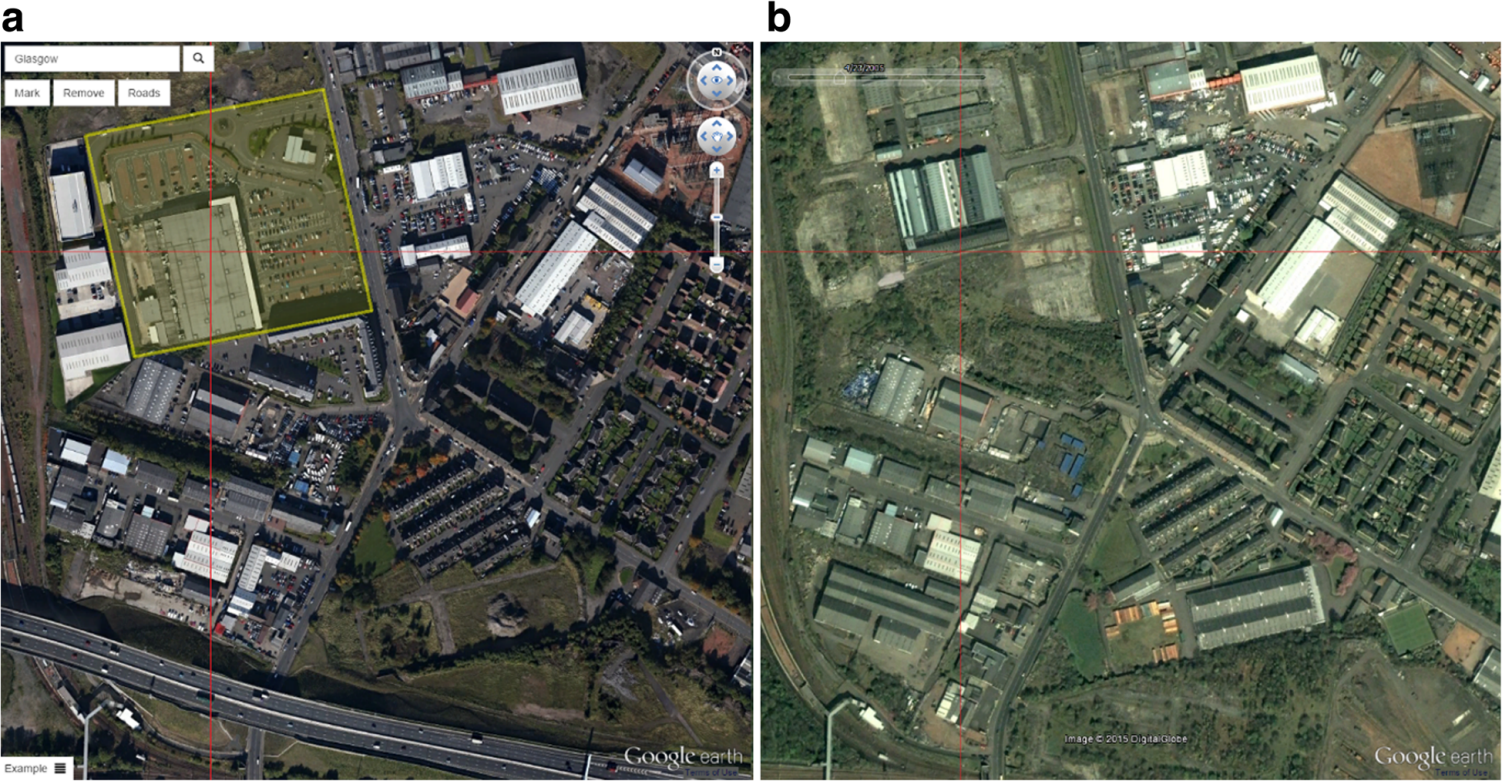
Example of environmental change identified by comparing two satellite images: (**a**) 2015; (**b**) 2005. Polygon outlined in yellow denotes one example of a building that has been demolished and a new building constructed in its place (for simplicity, other changes in this image have not been marked). The new motorway can also be seen at the bottom left of the image. Source: Google Earth

We developed bespoke software to display side-by-side Google Earth satellite images of the same location taken at different times, using the time slider function. The software allowed the operator to zoom the images and move them in tandem, manually comparing the two frames with the aid of overlaid cross-hairs. Areas of change between 2005 and 2015 were identified, which included the construction or demolition of buildings and the loss or gain of green space (Fig. 2). These areas were delineated with a polygon (multi-sided shape).

**Fig. 3:**
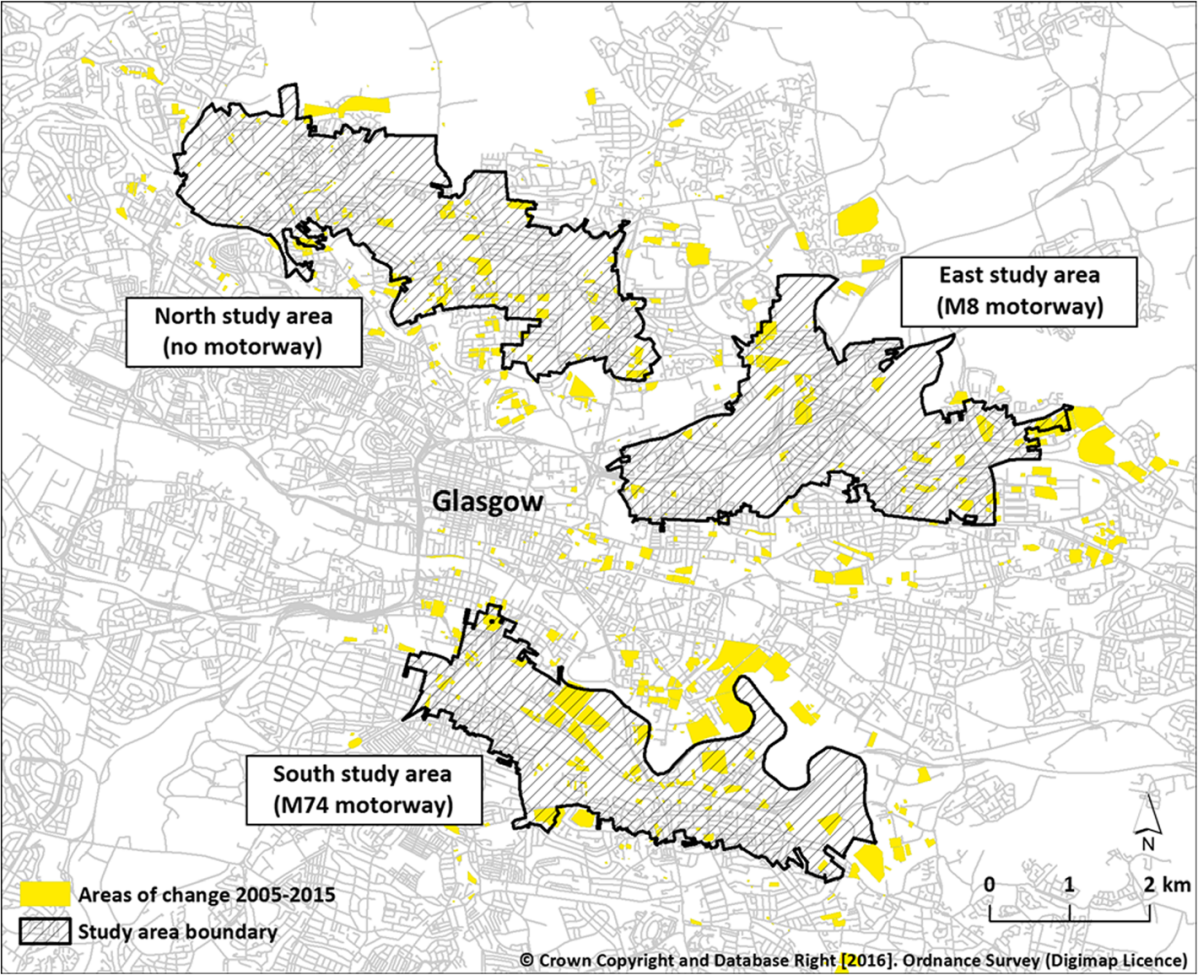
Areas of change within and surrounding the study areas in Glasgow, Scotland. . Crown Copyright and Database Right [2016]. Ordnance Survey (Digimap Licence)

Using this software, we identified all visible changes occurring between 2005 and 2015 in each of the three study areas and extending to approximately 1 km beyond their boundaries (Fig. 3). As expected, substantial changes had occurred in all three study areas. We did not characterise each change; we simply noted that change had occurred. This allowed us to explore the impacts of change per se regardless of the associated value of that change to local residents. For example, our exposure measure included both gain and loss of green space, and both building and demolition, all of which were coded as change. Furthermore, we did not identify the general (e.g. residential, retail or industrial) or specific (e.g. new supermarket, demolished sports club) features of the identified changes. The carriageway of the new M74 motorway was not included, although we did include the demolition and construction of buildings and roads nearby.

**Fig. 4:**
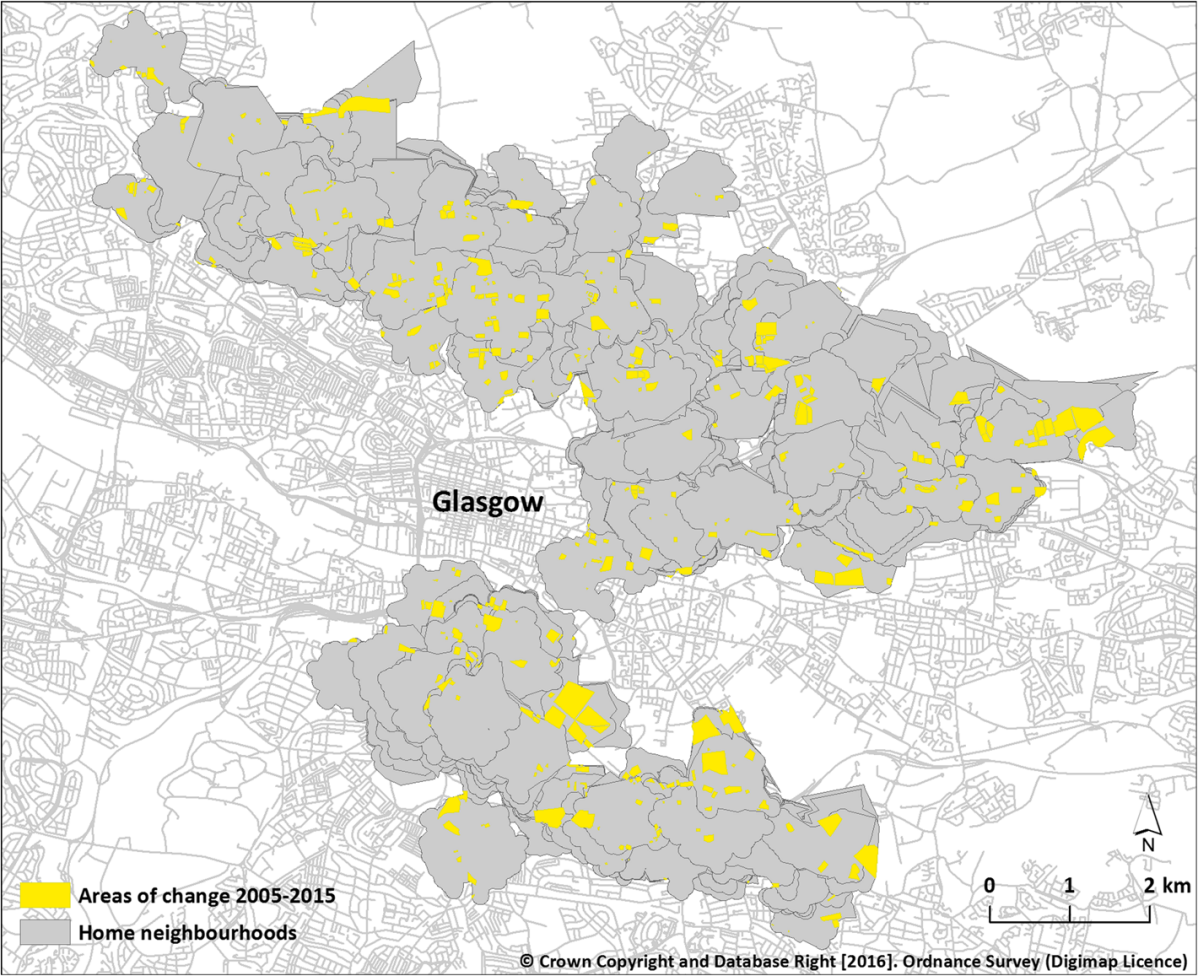
Areas of change within participants’ neighbourhoods. Crown Copyright and Database Right [2016]. Ordnance Survey (Digimap Licence)

**Fig. 5:**
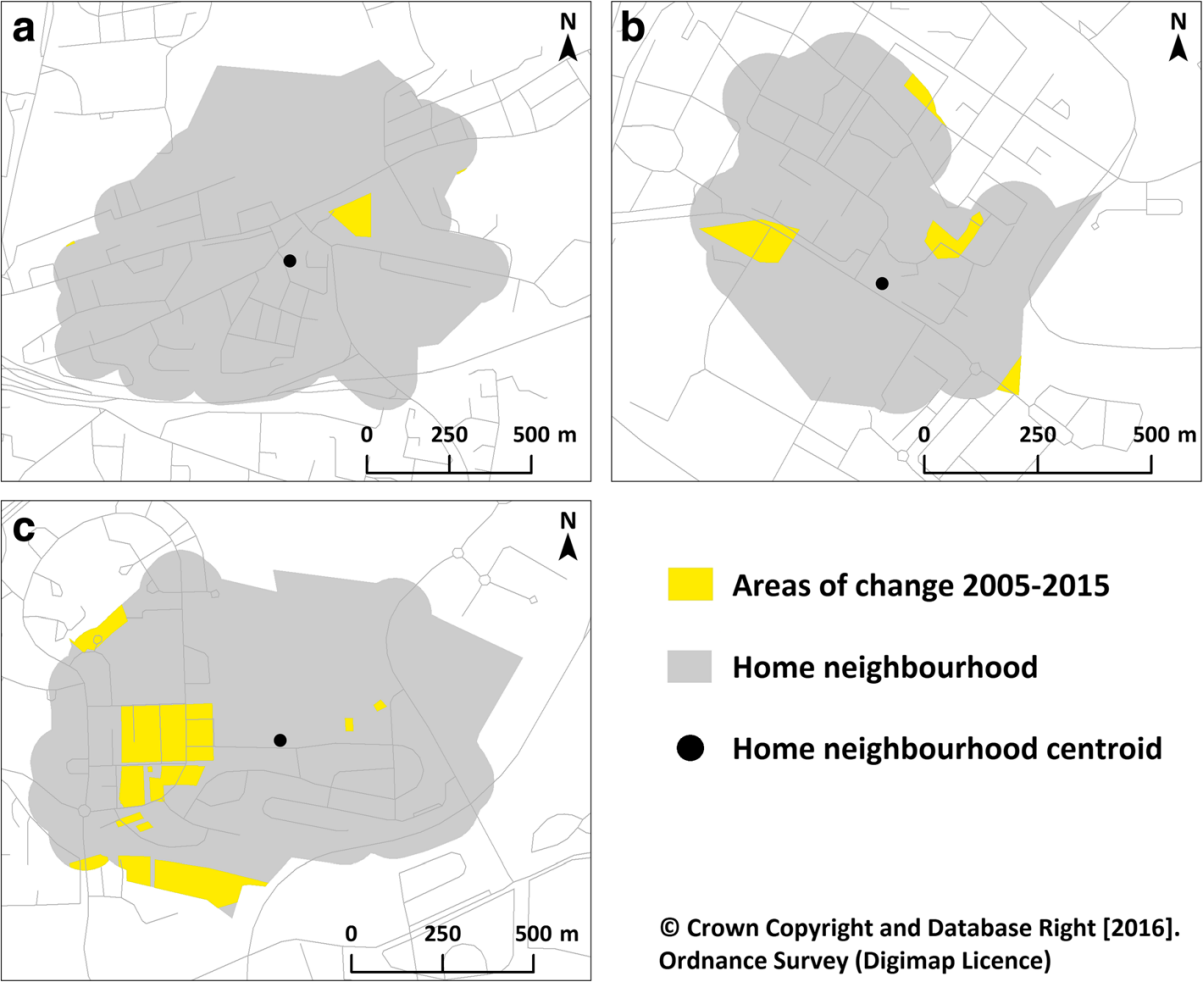
Examples of individuals’ neighbourhoods experiencing different levels of change in which approximately (**a**) 1%, (**b**) 5%, and (**c**) 10% of the neighbourhood built environment changed between 2005 and 2015. Crown Copyright and Database Right [2016]. Ordnance Survey (Digimap Licence)

Following this, polygons were imported into a GIS (ArcGIS v10.1, ESRI), and the area in square metres of each polygon was calculated. Using each participant’s home address, we then delineated their neighbourhood which we defined using an 800 m pedestrian network buffer around the weighted population centroid of the unit postcode, similar to definitions used in other recent studies [29]. Finally, we identified the percentage of the area within each neighbourhood that had changed (Fig. 4). This gave an indication of the general amount of change occurring in each individual’s neighbourhood, whereby higher values represented a greater amount of change. A unit change in this exposure represented an increase of one percentage point. Examples of neighbourhoods experiencing different amounts of change can be found in Fig. 5.

### Analysis

Descriptive information on the longitudinal cohort was generated for baseline and follow-up characteristics. We explored changes over time, and differences between the longitudinal cohort and the remainder of the baseline sample lost to follow-up, using one-way analysis of variance (ANOVA), t and chi-squared tests.

Using linear regression models, we assessed the relationship of exposure to change in the neighbourhood built environment with change in (a) PCS-8 and (b) MCS-8. Covariates that we hypothesised could confound the association between place of residence and wellbeing, and that have been considered in other similar research, were added in steps. Model 1 was unadjusted; model 2 was adjusted for age and sex; model 3 was adjusted for the variables in model 2 plus home ownership, car ownership, working status, perceived financial strain, presence of a chronic condition and years lived in the local area; and the maximally adjusted model (model 4) was adjusted for the variables in model 3 plus the baseline value of the outcome of the model in question (PCS-8 or MCS-8). We did not impute data due to less than 6% missing values for all variables.

Following this main analysis, we tested all maximally adjusted models for interactions with baseline perceived financial strain, presence of a chronic condition, and years lived in the local area, characteristics which we hypothesised could moderate this relationship. Additionally, we tested all maximally adjusted models for an interaction with study area (new motorway, existing motorway and no motorway) to examine whether the outcomes of exposure to general neighbourhood change differed in those additionally exposed to motorway infrastructure. If interaction terms were statistically significant, we ran stratified analyses to elucidate their interpretation, removing the stratification variable as a covariate but retaining all other covariates described above. For all analyses, we set the alpha at 0.05.

## Results

### Response, retention and drop-out

One thousand three hundred forty-five completed surveys were returned at baseline in 2005. After accounting for 676 survey packs that could not be delivered and were returned to sender by the Royal Mail, the response rate was 16.1%. At follow-up in 2013 (8 years later), 365 of these participants, or 27% of the original baseline sample, were successfully contacted and returned a postal survey.

Compared to the rest of the baseline sample, cohort participants were statistically significantly more likely to be men, to own a home or a car, to be working or studying, and to describe themselves as financially “comfortably off”, though there were no differences for age, time lived in the local area or presence of a chronic condition.

### Descriptive characteristics

Descriptive characteristics of the cohort can be found in Table 1, and are presented by level change in the neighbourhood built environment in Additional file 1. Two hundred seventy-three of the 365 participants (75%) lived at a unique postcode. The sample was predominantly female, with approximately 60% of respondents reporting owning a home or car and being engaged in work or study at baseline, at which time participants were on average aged 50 years and had lived in the local area for 18 years. Approximately 40% of participants reported having a chronic condition, and around half reported that they had to be careful with money. The mean score for PCS-8 was 47.4 (standard deviation [SD] 11.0) and for MCS-8 was 45.5 (SD 11.1) at baseline.

**Table 1:** Characteristics of the cohort

Variable	Baseline (2005)		Follow-up (2013)	
	n	mean (SD) / %	n	mean (SD) / %
Age (years)	360	50.4 (13.6)	363	58.5 (13.6)^b^
% male	361	43.5	363	44.4
% home ownership	360	61.1	363	62.5
% car ownership	361	58.5	362	60.5
% working^a^	359	58.5	364	48.1^b^
% with chronic condition	360	38.9	361	47.9^b^
% perceived financial strain	361		361	
Quite comfortably off		11.9		12.5
Can manage without difficulty		20.2		24.4
Have to be careful with money		52.9		47.1
Find it a strain to get by		15.0		16.1
Years lived in local area	365	18.3 (15.3)	362	24.9 (16.6)^b^
SF-8 PCS-8	352	47.4 (11.0)	360	45.9 (11.7)^b^
SF-8 MCS-8	352	45.5 (11.1)	360	46.4 (11.1)

*n* = 365, *n* number, *SD* standard deviation, *SF-8 MCS-8* SF-8 mental component summary score, *SF-8 PCS-8* SF-8 physical component summary score

^a^In paid employment (full or part-time), full-time student, or undertaking voluntary work

^b^Significant difference between time points (*p* < 0.05)

Changes in these characteristics over time were consistent with an ageing cohort. In the whole sample, there was a statistically significant reduction in physical wellbeing over time (1.5 unit reduction), but no statistically significant changes in mental wellbeing. Exposure to change in the neighbourhood built environment varied widely between 0% and 57%, with a median value of 3% (interquartile range = 6). Additional file 1 indicated a trend toward a greater level of change in the neighbourhood built environment in lower socioeconomic individuals.

### Longitudinal association between exposure to change in the neighbourhood built environment and wellbeing

The results of the multivariable linear regression models are displayed in Table 2. For each 1% increase in change in the built environment, participants experienced, on average, a 0.13 point reduction in physical component summary scores (PCS-8: -0.13, 95% CI -0.26 to 0.00) and a 0.16 reduction in mental component summary scores (MCS-8: -0.16, 95% CI -0.31 to − 0.02) over time. These associations became statistically significant in maximally adjusted models after adjustment for baseline wellbeing. Visual inspection of the distribution of model residuals suggested the assumptions of linear regression had been satisfied. A Moran’s I test of the model residuals was not statistically significant, indicating that spatial autocorrelation was not an issue.

**Table 2:** Longitudinal associations between exposure to change in the neighbourhood built environment and change in SF-8 physical and mental component summary scores, 2005–2013

	Beta coefficient (95% CI)							
Exposure	Outcome: SF-8 physical component summary score (PCS-8)							
	n	Model 1	n	Model 2	n	Model 3	n	Model 4
1% change to built environment in neighbourhood	326	−0.10 (−0.23, 0.04)	324	−0.10 (− 0.23, 0.04)	314	− 0.10 (− 0.24, 0.04)	314	−0.13* (− 0.26, 0.00)
	Outcome: SF-8 mental component summary score (MCS-8)							
	n	Model 1	n	Model 2	n	Model 3	n	Model 4
1% change to built environment in neighbourhood	326	−0.13 (− 0.30, 0.04)	324	− 0.12 (− 0.28, 0.05)	314	−0.16 (− 0.32, 0.01)	314	−0.16* (− 0.31, − 0.02)

*CI* confidence interval, *n* number

*Significant at *p* < 0.05

Model 1 is unadjusted

Model 2 is adjusted for age and sex

Model 3 is adjusted for variables in model 2 plus home ownership, car ownership, working status, perceived financial strain, presence of a chronic condition and years lived in the local area

Model 4 is adjusted for variables in model 3 plus baseline value of the outcome of the model in question

### Moderation of the association between exposure to change in the neighbourhood built environment and wellbeing

**Fig. 6:**
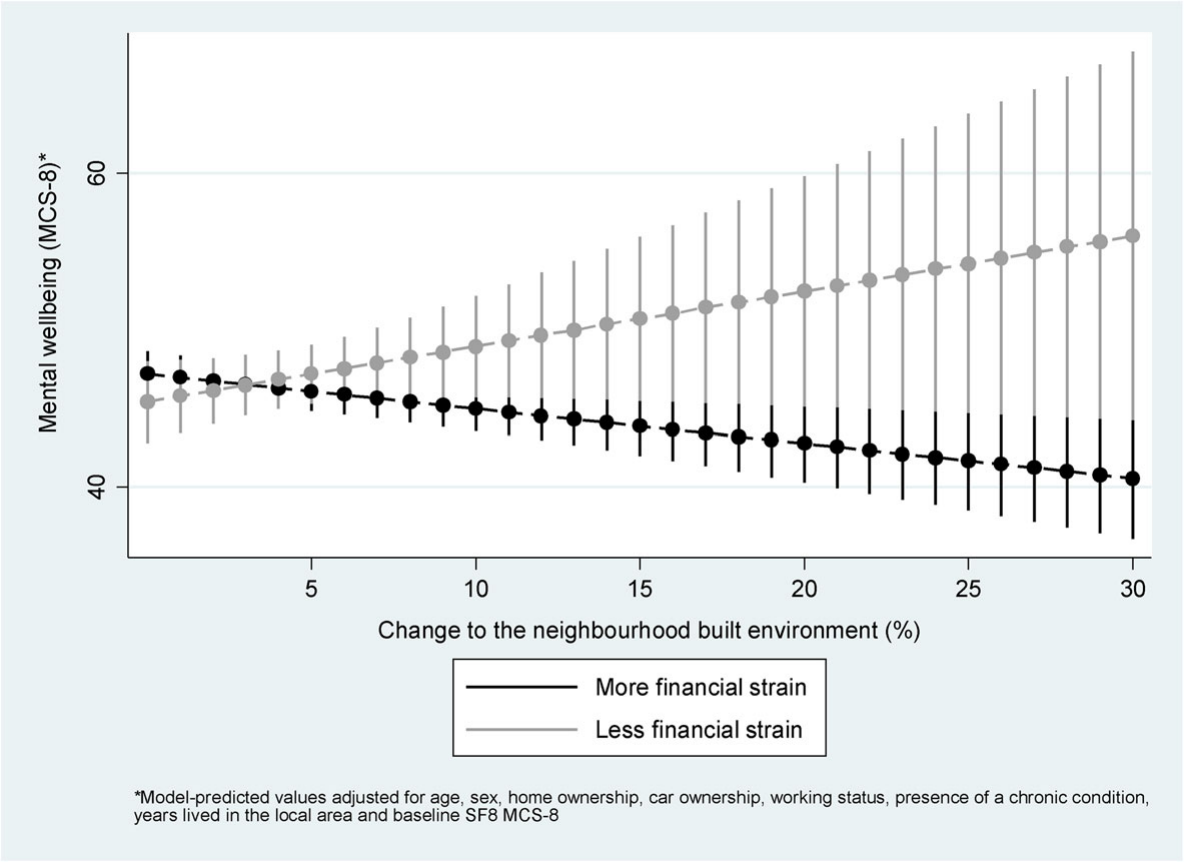
Statistically significant moderation of the relationship between exposure to change in the neighbourhood built environment and mental wellbeing by baseline perception of financial strain

For mental wellbeing, a statistically significant (*p* = 0.017) interaction by baseline perceived financial strain was found. Stratified analysis indicated that in participants experiencing greater financial strain, those living in neighbourhoods with a greater amount of change in the built environment experienced significantly reduced mental wellbeing over time compared to those living in neighbourhoods with less change (MCS-8: -0.22, 95% CI -0.39 to − 0.06), a slightly larger coefficient than that found in the main analysis. However, in participants experiencing less financial strain, those living in neighbourhoods with a greater amount of change in the built environment experienced significantly improved mental wellbeing over time compared to those living in neighbourhoods with less change (MCS-8: 0.38, 95% CI 0.04 to 0.72), a reversal of the relationship found in the main analysis (Fig. 6).

To test the robustness of this finding, we conducted a post-hoc analysis creating another binary variable indicating participants who had less financial strain at both time points (i.e. persistently less strain; *n* = 84) versus all other combinations (i.e. persistently greater or mixed pattern of financial strain; *n* = 281). A statistically significant (*p* = 0.046) interaction by this new variable was found, with the pattern of outcomes mirroring the moderation analysis described above. In participants experiencing persistently less financial strain, those living in neighbourhoods with a greater amount of change in the built environment experienced significantly improved mental wellbeing over time (MCS-8: 0.41, 95% CI 0.02 to 0.79). In those experiencing other combinations of financial strain, those living in neighbourhoods with a greater amount of change in the built environment experienced significantly reduced mental wellbeing over time (MCS-8: -0.20, 95% CI -0.36 to − 0.04). Finally, we found no significant interactions by baseline presence of a chronic condition or years lived in the local area.

### Moderation of the association between exposure to change in the neighbourhood built environment and wellbeing by motorway exposure

For mental wellbeing, a statistically significant (*p* = 0.042) interaction was found by study area for the study area containing the new M74 motorway. Stratified analysis indicated that participants in this area living in neighbourhoods with a greater amount of change in the built environment experienced significantly reduced mental wellbeing over time compared to those living in neighbourhoods with less change (MCS-8: -0.18, 95% CI -0.34 to − 0.03).

## Discussion

### Main findings

We found some evidence that living in neighbourhoods experiencing higher levels of change in the built environment was associated with worsening physical and mental wellbeing in local residents. However, this overall pattern of findings was not distributed uniformly; for mental wellbeing, there was a larger reduction among those reporting greater levels of financial strain. Financial security reversed the relationship, with higher levels of neighbourhood change associated with improvements in mental wellbeing in those reporting greater financial security. It may be that the purpose, drivers and nature of environmental changes fundamentally differ between wealthier and less wealthy neighbourhoods, explaining the associations we found. Finally, we found that the negative association between exposure to general change and mental wellbeing was heightened in those additionally exposed to another specific change in the built environment – the construction of a new motorway.

### Strengths and limitations

In this proof of concept study, we devised a novel and simple method for identifying and quantifying the degree of physical environmental change by using satellite imagery in the public domain. This contributes to efforts in the field to ‘longitudinalise’ contextual information [30]. In addition, we used an extensively validated tool to assess wellbeing. We then found novel, statistically significant associations between exposure to change in the neighbourhood built environment and individual-level change in wellbeing over time in a longitudinal cohort, which differed by socioeconomic status. This study contributes to an accumulating body of research on how changes to the built environment affect health and health inequalities [31].

We also acknowledge the study limitations. It is likely that self-selection bias occurred, and we make no claim that our sample were representative of the general local population. Initial response to the postal survey was low (16%), and attrition of the cohort over 8 years was high (73%), but comparable to other similar studies [10, 32]. In addition, our measure of neighbourhood environmental change was not validated, and because it was developed later in the project there was a slight mismatch between the study period (2005–13) and the timescale of the satellite images (2005–15). The use of 800 m buffers to define the neighbourhood is consistent with other studies, but research indicates that people roam considerably further in their daily lives [29]. However, a gold-standard method of assessing neighbourhood environmental change does not exist. As such, this was an exercise in pragmatism to demonstrate the potential of using satellite imagery as one form of “ragged evidence” [33], to contribute to learning in the wider field of environmental interventions, and to set the scene for future research.

Our assessment of total change was a simplified approach to understanding what is undoubtedly a complex relationship between the environment and health. In particular, the lack of specificity makes it difficult to identify causal mechanisms between exposure and outcome, as we were not able to characterise changes and therefore get a sense of which changes were ‘positive’ or ‘negative’. The characterisation of changes identified from satellite imagery is therefore an important avenue for future research. However, this study was intended to be hypothesis generating rather than definitive, and our previous research highlights some possible mechanisms, which are discussed further in Section 4.4. Finally, the possibility of non-independent observations, unmeasured confounding and the specificity of findings to the context in which they occurred are core, but mostly unavoidable, limitations of research in this area.

### Comparison with other studies

Participants living in neighbourhoods with a greater amount of change in the built environment experienced reduced physical (PCS-8: -0.13, 95% CI -0.26 to 0.00) and mental (MCS-8: -0.16, 95% CI -0.31 to − 0.02) wellbeing over time compared to those living in neighbourhoods with less change. Although these coefficients may appear comparatively small, they represent estimates of the difference in wellbeing between participants living in neighbourhoods that differed in change by a single percentage point. It may be more meaningful to compare neighbourhoods that differ by a larger ‘dose’ of change, for example 5% or 10% (see Fig. 5). For these comparisons, the estimated differences in physical wellbeing are − 0.65 and − 1.30 PCS-8 units respectively, and for mental wellbeing are − 0.80 and − 1.60 MCS-8 units respectively. To place these changes in context, they are modest compared to previous clinical research in headache sufferers that observed a 3.0 unit (physical wellbeing) and 3.3 unit (mental wellbeing) reduction in patients experiencing reduced overall quality of life [25]. However, our findings occurred in a non-clinical, community-based context, in which detrimental effects might accumulate over time.

The current study evaluated general change, and is not an assessment of the impacts of urban renewal. However, we highlight previous urban renewal research as both exposures incorporate elements of physical change. Previous studies have mostly found either no change or modest improvements in wellbeing following targeted urban renewal [2, 9–13]. In particular, another study conducted in Glasgow evaluating the impacts of neighbourhood demolition and renewal found a statistically significant increase in mental wellbeing in participants receiving housing improvements relative to controls, using the SF-12, another derivation of the SF-36 (2.4, 95% CI 0.0 to 4.8) [10]. In that study, physical deterioration and demolition of neighbourhoods did not appear to adversely affect residents’ wellbeing [10], but specific types of housing improvements (such as a new, secure front door) had mostly positive impacts on mental wellbeing [11], and higher total monetary investment in renewal led to greater improvement in mental wellbeing over 5 years compared with lower investment (4.3, 95% CI 0.3 to 8.2) [12].

Previous research also suggests that urban renewal can narrow (or at least not exacerbate) existing inequalities in health or wellbeing between lower and higher socioeconomic groups [9, 12–14]. Those renewal packages targeted specific improvements to the built environment and investment was allocated according to need. In our study, we examined general change to the built environment, and our findings were more similar to previous work suggesting that individuals from lower socioeconomic groups benefit less from interventions aimed at the general population [34, 35], with the important distinction that in our study the changes were associated with a harmful trend in lower socioeconomic groups.

### Implications for research

Several issues may be of interest to researchers in the field. Firstly, our exposure measure could be described as broad, or more pejoratively as crude. However, other researchers evaluating changes in the built environment have used similarly broad exposures, where the specific type of environmental change occurring was not captured [12, 13]. As described in Egan et al. [12], the use of a broad exposure may allow us to move beyond the ‘form’ of a particular environmental change to focus on the ‘function’ of that change [36], though we do not claim to have identified the specific function in this preliminary analysis.

A related issue is the challenge of identifying plausible causal mechanisms by which these broad exposures may be related to increases or decreases in wellbeing. In our larger mixed-method study examining the impacts of living near a new urban motorway, qualitative research gave insights into the causal recipe by which exposure to a motorway was associated with reductions in wellbeing. This work suggested that noise and air pollution had contributed to worsening wellbeing in those living very close to the new road [23]. In the current analysis, much of our neighbourhood change exposure was comprised of demolition or construction and thus it is plausible that similar mechanisms were involved. In addition, the qualitative study indicated that the social fabric of communities was also undergoing a rapid change, which many related to successive waves of immigration. In some cases, this had introduced a sense of fraying social cohesion in the community. Albeit conducted in only one of the three study areas that comprised the sample for the current analysis, these observations highlight the contribution of changes in both the physical and social environment, and suggests that both mechanisms could have been operating in the current study.

### Implications for policy and practice

There is currently little clear public health evidence about the impacts of targeted urban renewal, or more general changes to the built environment, to guide policy and planning. Change may bring new opportunities to neighbourhoods and communities, but it may be that individuals require the financial resources to capitalise on these opportunities. For urban renewal specifically, some have suggested that this can increase the cost of living [37] and contribute to gentrification of the area [38], which might have negative impacts on those in an already financially precarious situation, and eventually lead to their displacement from the area. On the other hand, research suggests that some environments can narrow socioeconomic inequalities in health. Access to recreational or green areas is a key feature of these so-called ‘equigenic’ environments [39], though some suggest that improvements in access to urban green space can also accelerate gentrification [40].

## Conclusions

We found some evidence that living in neighbourhoods experiencing high levels of physical change worsened wellbeing in local residents. However, we found a stronger negative relationship in those with lower levels of financial security and a positive relationship in those with higher levels of financial security. This is one of few studies exploring the longitudinal relationship between the environment and health.

## Additional file

Additional file 1: **Table S1.** Baseline characteristics of the cohort by level of change in the neighbourhood built environment. A supplementary table showing baseline characteristics of the analysis sample. (DOCX 13 kb)

## Abbreviations

ANOVA: Analysis of variance
CI: Confidence interval
GIS: Geographic information system
MCS-8: Mental component of the SF-8 scale
n: Number
PCS-8: Physical component of the SF-8 scale
SD: Standard deviation
UK: United Kingdom
