# Supplementary material for: Longitudinal association between change in the neighbourhood built environment and the wellbeing of local residents in deprived areas: an observational study
Source: BMC Public Health. 2018 Apr 24;18:545. doi: 10.1186/s12889-018-5459-9 (PMC5921539; doi:10.1186/s12889-018-5459-9)
Supplement: Supplementary file 1 — Table S1. Baseline characteristics of the cohort by level of change in the neighbourhood built environment. A supplementary table showing baseline characteristics of the analysis sample. (DOCX 13 kb) [file 12889_2018_5459_MOESM1_ESM.docx]

Additional file 1

Table 1: Baseline characteristics of the cohort by level of change in the neighbourhood built environment

| **Baseline variable** | **Change in neighbourhood build environment** | | | | | |
| --- | --- | --- | --- | --- | --- | --- |
|  | **0 to 5% change** | | **>5% to 10% change** | | **>10% change** | |
|  | *n* | *mean (SD) / %* | *n* | *mean (SD) / %* | *n* | *mean (SD) / %* |
| Age (years) | 213 | 50.9 (13.5) | 83 | 50.5 (12.7) | 42 | 54.5 (13.9) |
| % male | 213 | 42.7 | 83 | 45.8 | 43 | 41.9 |
| % home ownership | 213 | 63.9 | 83 | 55.4 | 42 | 50.0 |
| % car ownership | 213 | 60.1 | 83 | 51.8 | 43 | 48.8 |
| % working* | 213 | 57.3 | 81 | 56.8 | 43 | 46.5 |
| % with chronic condition | 211 | 36.5 | 84 | 46.4 | 43 | 53.5 |
| % perceived financial strain | 213 |  | 83 |  | 43 |  |
| Quite comfortably off |  | 13.2 |  | 10.8 |  | 2.3 |
| Can manage without difficulty |  | 22.1 |  | 15.7 |  | 18.6 |
| Have to be careful with money |  | 53.1 |  | 55.4 |  | 51.2 |
| Find it a strain to get by |  | 11.7 |  | 18.1 |  | 27.9 |
| Years lived in local area | 215 | 18.5 (15.6) | 85 | 18.4 (14.7) | 43 | 22.1 (15.9) |
| SF-8 PCS-8 | 207 | 47.9 (10.8) | 82 | 46.0 (11.5) | 41 | 44.0 (11.6) |
| SF-8 MCS-8 | 207 | 45.9 (10.8) | 82 | 44.6 (12.8) | 41 | 43.6 (10.4) |

n – number; SD – standard deviation; SF-8 MCS-8 – SF-8 mental component summary score; SF-8 PCS-8 – SF-8 physical component summary score

*In paid employment (full or part-time), full-time student, or undertaking voluntary work
